# Unexpected Result of Hendra Virus Outbreaks for Veterinarians, Queensland, Australia

**DOI:** 10.3201/eid1801.111006

**Published:** 2012-01

**Authors:** Diana H. Mendez, Jenni Judd, Rick Speare

**Affiliations:** James Cook University, Townsville, Queensland, Australia

**Keywords:** veterinarian, veterinary profession, Hendra virus, horses, infection control, workplace health and safety, virus, zoonotic, zoonoses, viruses, Australia

## Abstract

A qualitative study of equine veterinarians and allied staff from Queensland, Australia, showed that veterinarians are ceasing equine practice because of fears related to Hendra virus. Their decisions were motivated by personal safety and legal liability concerns.

In the mid-1990s, Hendra virus (HeV) emerged as a new pathogen that spilled over from bats to horses to humans (*1**,**2*). All 7 cases of HeV infection among humans in Australia occurred in Queensland. Five of these cases involved equine veterinary personnel who conducted routine necropsies or endoscopies; 3 of the 5 cases were fatal (*2**–**6*). In Australia, equine clinical services are mostly delivered by veterinarians working in private practice. The 3 deaths prompted government and veterinary professional agencies to promote the overhaul of infection-control measures in veterinary practice (*3**,**4*) and increase auditing of veterinary infection-control strategies in private equine practice by Workplace Health and Safety Queensland (*7*). In 2011, HeV outbreaks multiplied throughout Queensland and New South Wales, and samples from a dog were positive for HeV (*8**,**9*).

With the approval of the James Cook University Human Ethics Committee (permit H3513), we interviewed veterinarians and allied staff from veterinary practices with the aim of capturing the HeV-related infection-control and workplace health and safety issues faced by equine practices. We report on 1 unexpected emerging issue: the departure of veterinarians from equine practice as a result of HeV outbreaks.

## The Study

During 2009–2010, we conducted face-to-face, in-depth interviews with 21 veterinarians and allied staff from 14 equine and mixed private veterinary practices from a range of urban and rural areas between Cairns and Brisbane, Queensland, Australia (Table 1) (*10*). We asked a series of open-ended questions to determine what HeV-related infection-control and workplace health and safety issues confront equine practices (Table 2). Interviews were recorded, transcribed, and analyzed for themes.

**Table 1:** Location of participants in a study of Hendra virus–related safety issues faced by equine practices, Queensland, Australia, 2009–2010*

Zone, category	No. (%) participants
Metropolitan zone	
Capital cities	3 (14.30)
Population >100,000	6 (28.55)
Rural zone	
Population 25,000–99,999	6 (28.55)
Population 10,000–24,999	0
Population <10,000	3 (14.30)
Remote zone	
Population >4,999	3 (14.30)
Population <5,000	0

*Location zones and categories are according to the Australian Rural, Remote and Metropolitan Areas classification system (*10*).

**Table 2:** Demographic characteristic of participants in a study of Hendra virus–related safety issues faced by equine practitioners, Queensland, Australia, 2009–2010

Study participants	No. (%)	Age, y (range)*	Years since graduation* (range)†	% Time spent doing equine work* (range)‡	Distribution by job title, no. (%)			
					Principal veterinarian	Partner/associate or employee veterinarian	Veterinary nurse	Practice manager
Female	8 (38.1)	35.8 (31–48)	13.1 (4–27)	30.4 (2–95)§	1 (4.8)	5 (23.8)	2 (9.5)	0
Male	13 (61.9)	48.5 (28–63)	22.9 (4–40)	52.1 (2–100)¶	9 (42.8)	3 (14.3)	0	1 (4.8)
Total	21 (100.0)	42.2 (28–63)	19.0 (4–40)	47.3 (2–100)	10 (47.6)	8 (38.1)	2 (9.5)	1 (4.8)

*Average. †The practice manager interviewed was not a veterinarian and did not wish to supply this information. ‡Self-reported. §One female participant did not provide this information. ¶The 1 participant who was a practice manager but not a veterinarian had not spent any time with animals and therefore was not included.

Of the 20 veterinary professionals interviewed, 12 (60%) had dealt with >1 suspected cases of HeV, and 7 (35%) had dealt with a confirmed case of HeV. Of the 18 veterinarians interviewed, 4 (22%) reported having ceased equine practice, and as many as 8 (44%) knew of >1 colleagues who had done so in the previous 12 months. The decisions to quit were mostly motivated by the HeV-related fear for personal safety and legal liability.

Under the current Queensland legislation governing private businesses, private veterinarians are responsible for the safety of all persons in their workplace, both in the clinic and the field (*11*). Ten (47.6%) of the study participants were principal veterinarians (Table 1) who carried the highest degree of legal responsibility within their veterinary practice; they were quite concerned about their HeV-related legal liability. Four of these principal veterinarians reported ceasing equine practice because of the difficulty in enforcing infection control–related workplace health and safety compliance among their staff, because the logistical outlay of bringing change to their practice was too costly, or both. One participant declared, “The HeV situation was the last straw that made us stop equine practice…. We put it in the too hard basket.” Their fear of prosecution became too big a threat for their business. However, ceasing this high-risk activity does not result in improved infection-control standards.

Principal veterinarians from other practices preferred to personally deal with all equine patients, thus taking the highest risk themselves rather than putting their staff at risk or not providing the service. In some instances, staff and principal veterinarians resorted to working in suboptimal personal safety conditions to fulfill their legal and ethical responsibility to their patients and clients, thus jeopardizing the legal situation. As one participant pointed out, “Veterinarians usually end up with less authority… taking the risk out of concern for the welfare of the horse.” Veterinarians have a legal right to refuse service if safety is compromised; however, this would mean forfeiting immediate and future income through the loss of a client(s) and, possibly, reputation. In such instances, the staff and the business remain safe, but the principal veterinarian may not, and the overall standards of infection control within the practice do not improve.

Up to 6 (60%) of the interviewed principal veterinarians had embraced the need for improvement of infection-control practices and had made major changes to their protocols and premises, but they felt that the best level of compliance would not be legally protective because of the unpredictable character of the veterinary work environment. Another participant expressed concern over this legal uncertainty: “You still have to worry about what might occur out of the blue…. With workplace health and safety we are very aware that complying is often not enough if an incident occurs.” In this scenario, although safety improvement is achieved, the legal risk remains.

Those participants still in equine practice also expressed concern over the consequences that the loss of skilled equine veterinarians would have on the profession and their practice. One participant said, “… this might introduce problems of gaps in the welfare of animals. Vets will need to refer animals.” The lack of equine specialists would increase demands on the remaining equine veterinarians, who would have to further extend their already overstretched time and resources: they would work longer hours, travel farther to provide services, and be unable to reach sick horses in remote locations or to have them tested in a timely fashion. Participants still in equine practice considered that all these factors made working with horses less safe. Indeed, several studies showed that across a wide range of sectors, working >60 hours/week increased the risks for occupational injury and illness (*12**–**14*). Furthermore, several study participants reported that some colleagues now choose to only provide services to healthy animals and refuse to treat sick horses. A participant described this as choosing the “easy safe money” over the “hard dangerous money.” This choice was creating resentment among members of an otherwise tight-knit veterinary community. Over time, resentment could jeopardize professional networking, which seems to play an essential role in disseminating clinical and safety information among veterinarians.

Although this study did not measure the overall effect of the decreased number of veterinarians who treat equids in Queensland, participants viewed the decrease as a major source of increased occupational risk for the remaining equine practitioners. If this trend is sustained, more private veterinarians may cease equine practice. Other participants no longer regarded themselves as equine practitioners and declared that they had ceased equine practice; however, they later admitted to still regularly treating horses. Their “official” departure from equine practice would increase their safety and legal risks because they might miss program updates on equine health information or infection-control improvement. It is also possible that the perception of increased risk may adversely influence the decision by younger veterinarians to pursue work in equine practice, thereby jeopardizing the normal replacement of the existing pool of aging equine practitioners. One parallel was the effect of severe acute respiratory syndrome. Overall, 35% of severe acute respiratory syndrome–related deaths were in health care workers. Some workers refused to go to work and others adopted a heroic stance and continued to work, resulting in substantial medium-term psychological effects on the healthcare professionals (*15*).

## Conclusions

HeV remains a threat to the veterinary profession and public health in Australia. The experimental success of an HeV vaccine for horses was recently announced; if a vaccine becomes available, it may re-instill confidence in existing and future equine practitioners (*9*). However, the potential that emerging infectious diseases might dismantle the veterinary workforce should be considered when developing official strategies for the management of HeV outbreaks. Infection-control management guidelines and workplace health and safety regulations must consider the context in which services are feasibly delivered to the public and should be devised in consultation with the private veterinary professionals on the frontline of outbreaks.
